# First facts on the distribution of personal protective equipment during the coronavirus pandemic and facts revealed by medical entities in Brazil: a cross-sectional study

**DOI:** 10.1590/1516-3180.2021.0974.R2.03062022

**Published:** 2022-09-12

**Authors:** Mario Afonso Maluf, Rui Nunes

**Affiliations:** I MSc. Physician and Doctoral Student in Bioethics, Faculty of Medicine of the University of Porto (U. Porto), Porto, Portugal;; Psychiatrist, Santa Catarina State Hospital of Custody and Psychiatric Treatment, Florianópolis (SC), Brazil.; II PhD. Physician and Coordinator of the doctoral course in Bioethics, Faculty of Medicine of University of Porto (U. Porto), Porto, Portugal; Coordinator of the Doctoral Course of the Agreement between the University of Porto and Conselho Federal de Medicina, Brasília (DF), Brazil.

**Keywords:** Public health, COVID-19, Personal protective equipment, Bioethics, Human resources management, Quality of work life, Mysthanasia, Maslow scale

## Abstract

**BACKGROUND:**

The 2019 coronavirus pandemic (COVID-19) has revealed precarious public health conditions worldwide, where serious failures have occurred, similar to the distribution of personal protective equipment (PPE) to physicians in the government of Brazil.

**OBJECTIVE:**

The objective of this investigation was to prove through facts that there have been failures in the distribution of PPE to medical professionals within a reasonable timeframe.

**DESIGN AND SETTING:**

Through a cross-sectional study, we sought to identify the information and data on the subject of “Distribution of PPE” from the official sites of all the national and regional medical representative entities.

**METHODS:**

All medical representative entities, such as unions, councils, and federations, were identified by searching their existing websites, which were active on the World Wide Web, identifying facts, news, and official data regarding the supply of PPE on a daily basis and during the research period.

**RESULTS:**

It was evident from the identification of over 3,900 physician complaints and news reports that there was a failure to distribute PPE to medical professionals in Brazil over a reasonable period. Several physicians obtained PPE through the ruling of the courts.

**CONCLUSIONS:**

There was indeed a failure in the context of health service administration, which compromised the second level of the Maslow Scale, safety needs, and exposed these professionals to a greater risk than necessary, compromised the quality of work life, and directly compromised the doctor-patient relationship. The condition of the physicians cannot be forgotten during the COVID-19 pandemic.

## INTRODUCTION

The public management of health services in Brazil can be described as compromised or deficient. In addition to issues with the quality of the services and supply of personal protective equipment (PPE) during the coronavirus disease 2019 (COVID-19) pandemic, poor management has had a negative impact on the quality of work life (QWL) of the medical professionals.

The pandemic officially began in Brazil on February 26, 2020,^1^ with the first case of a 61-year-old man, as confirmed by the Ministry of Health. The patient was a businessman and resident of the city of São Paulo, who arrived from the Lombardy region in Italy. According to the health officials, he was unlikely to have been the first patient.^2^

The COVID-19 pandemic that began in Wuhan, a province located in the People’s Republic of China, affected almost every country worldwide, leading to a huge effort to meet the demands for health services and ultimately dramatically further compromising the economies of nations,^3^ and compromised the world economy by more than US$ 700 billion in 2020, exceeding the global financial crisis of 2008 by 60%.^4^

The impact of the COVID-19 pandemic will be marked in the main economies globally, and in developing countries where two-thirds of the world’s population lives, with strong pressure on their financial systems, demanding coordinated actions and help from everyone for the coming months.^5^

Out of respect for physicians who are fellow human beings, the state should not demand irresponsibly that these professionals risk their lives beyond the natural risk of the profession. Such an increase in risk would compromise the QWL and adversely impact the specific and necessary technical activities to be performed during the COVID-19 pandemic. The facts are connected to mysthanasia—silent, little discussed, and causing much less revolt than it deserves: a crime not yet typified in the Penal Code, which comes from Greek etymology (mys = unfortunate; thanathos = death; “unfortunate death”).^6^ It is a miserable, precocious, and preventable death. It is the death facilitated by the three levels of the government, through sustained poverty, lack of infrastructure, and minimum conditions to have a dignified life.^7^ Mysthanasia is observed to be part of the context of Bioethics.

The above is evidenced by the following statistics: from February 20, 2020, to June 27, 2020, a total of 19,037 physicians were infected, and 247 of these physicians eventually died.^8^ We extended this survey until the last day of August 2020.

In Brazil, where the public sector employs approximately 73.1% of medical professionals, this problem is more serious and the need for a true policy of effective management is evident. As already pointed out, the survey revealed that 21.6% of physicians worked only in the public sector, whereas 26.9% worked exclusively in the private sector. As there is an overlap, 51.5% of physicians work concurrently in the public and private spheres; it can be stated that 78.4% of physicians have ties to the private sector and 73.1% to the public sector.^9^

### Maslow scale

**Figure 1.**
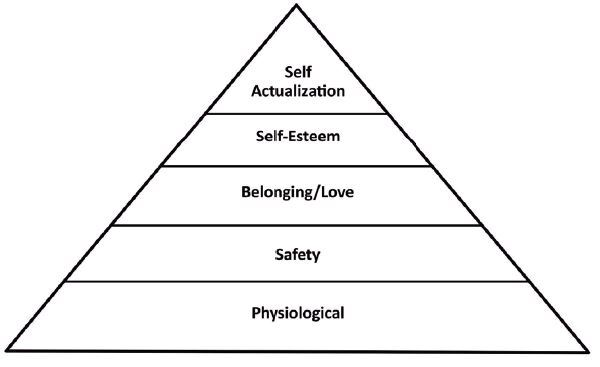
Maslow’s scale of needs.

Adapted from Ferreira et al.^12^

We chose Maslow’s classical, universal scale (Figure 1) as a reference for this study. The lack of PPE distribution to the medical professional class that is on the front line in the fight against the COVID-19 pandemic can lead to a higher exposure of medical professionals to the virus, resulting in a higher risk of pathology, that may be fatal. Thus, concerns about their own safety may lead to a state of mental imbalance in these professionals owing to a greater exposure to the risk of death. During this pandemic, several medical professionals experienced real health impairments, which led to hundreds of work absences, hospitalizations, and numerous deaths.^10^

The number of healthcare professionals, including physicians with COVID-19, and the incidence of death were significant in Brazil. According to the Ministry of Health, Brazil has had 31,790 confirmed cases of health professionals with COVID-19. A further 114,000 cases are currently under investigation. Brazil still has the highest rate of death among nurses worldwide because of the COVID-19 pandemic.^11^

The class of medical professionals has a more refined educational level, and the purpose of the profession is supported and grounded with a strong physician-patient relationship and bioethics. Perhaps these reasons have led medical professionals to remain systematically active, even with an increased risk of death. Within the scope of this sample, it is concluded that the Maslow’s Hierarchy of Needs, when applied to the work environment, presents an increasing trend of preference in relation to the level of education; that is, the longer the study time, the higher the Maslow scale, and the higher the needs of people.^12^

Regardless of the involvement of the second hierarchical (safety needs) level of the Maslow scale and their educational level, the physicians were inevitably exposed to an increased risk of death by COVID-19 due to the lack of PPE. Therefore, the sophistication of Maslow’s needs pertaining to the medical professional class, in proportion to their level of education and the existence of a strong physician-patient relationship, does not result in an absence, *sine qua non,* of their real safety needs. There is a widespread fear of the implications of disease and death. The commitment of the second level of the Maslow scale is due to facts such as complaints about safety and stability at work, the fear of being arbitrarily dismissed, not being able to plan a family budget due to a lack of guarantees regarding one’s permanence at work, and arbitrariness of the supervisor with respect to possible indignities to which the individual has to remain at work, his own physical safety in relation to the possible accidents at work, and more efficient and active medical care.^13^

The impairment of the level of safety experienced by the Brazilian medical professionals was evidenced by hundreds of complaints that arrived at representative institutions, which were also reported by the press. If the medical professionals had not felt threatened, there would not have been numerous complaints from all over the country.

The concern and care for the medical professionals compromised the second level of the Maslow Scale, Figure 1 and, consequently, the QWL. The quality of life (QOL) theory developed by Abraham Maslow’s human developmental perspective was presented.

A counterpoint to these statements is the issue of facts reported in this research, where the non-distribution of PPE by the states for the Brazilian medical class simply reached the basic level of the Maslow scale, which compromised their safety and that of the population.

### Medical Institutions that fought to get PPE

**Figure 2.**
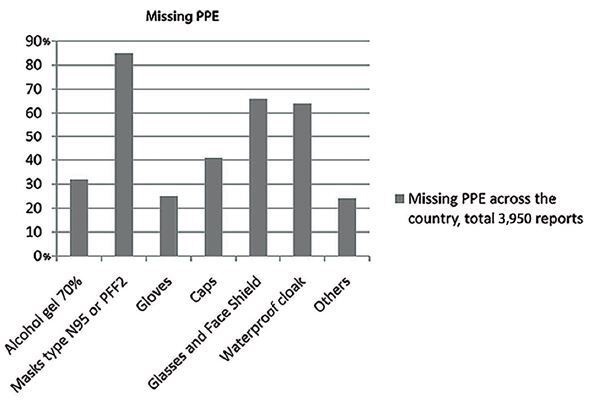
Missing personal protective equipment (PPE).

Adapted from Brazilian Medical Association.^15^

Institutions from all over Brazil, such as the Brazilian Medical Association (Associação Médica Brasileira, AMB), Federal Council of Medicine (Conselho Federal de Medicina, CFM), National Federation of Physicians (Federação Nacional dos Médicos, FENAM), and the Brazilian Medical Federation (Federação Médica Brasileira, FMB) are in solidarity with Brazilian physicians and other health professionals who contracted COVID-19.^14^ Specific pages for complaints were created, such as the one available at the Brazilian Medical Association (Associação Médica Brasileira, AMB).^15^ Additionally, several legal actions to obtain PPE, such as in the state of Paraná were undertaken.^16^ The same occurred with the regulative institution of the Brazilian medical class, the CFM, which initiated a series of specific recommendations, resolutions, and meetings with representatives of the federal government and made a direct link available on its official website to fill in the form referring to the complaints about the lack of PPE,^17^ as was also the case with AMB. The latter was the medical entity that received the most complaints regarding the poor distribution of PPE through the link https://amb.org.br/epi/, which made available a specific form for these complaints. The number of complaints was shown according to the table in Figure 2, during the period between February 1 and September 30, 2020.

Some medical unions, through their own link on their official website, redirected complaints regarding the lack of PPE to the official website of the AMB, where a dedicated form for these complaints was found.

There were difficulties in the acquisition and distribution of PPE in the initial phase of the epidemic in Brazil and practically worldwide. The initial inventories, acquisition capacity, and distribution of these PPEs were approached within the context of reasonableness.

### Prioritization in the distribution of PPE

The prioritization of health professionals to receive PPE is due to the fact that they are on the front line and are more exposed to COVID-19 than the general population. There is no controversy regarding the risk of contamination when working with pathogens, such as the severe acute respiratory syndrome coronavirus 2 (SARS-CoV-2) virus.^18^

The medical professional class is more likely to contract COVID-19 than the general population due to their exposure to the work environment, which includes direct contact with COVID-19 carriers. Our findings could help provide a greater context for previous cross-sectional reports from public health authorities, suggesting that 10–20% of SARS-CoV-2 infections occur among healthcare workers.^19^

Considering the need for the ethical distribution of PPE to medical professionals during the COVID-19 pandemic, there was an unprecedented increase in the consumption of equipment worldwide, which required efforts to meet the demand. It is difficult for clinicians to think about rationing PPE, particularly recognizing that decisions may expose some individuals to a greater risk of infection. However, if these decisions are to be made, they should be based on sound scientific and ethical principles, executed transparently and equitably, and subject to accountability. It is essential to minimize any moral residue from the decisions made during this pandemic such that once it is over, the task of rebuilding may be undertaken.^20^ An increase in the PPE supply in response to this new demand will require a large increase in PPE manufacturing, a process that will take time that several healthcare systems do not have, given the rapid increase in ill COVID-19 patients.^21^

The process of supply and distribution of PPE was not only restricted to the class of medical professionals, but also to all the health professionals who dealt directly with patients affected by COVID-19. It is essential that health workers use PPE during the COVID-19 pandemic; however, it is also essential to coordinate the supply chain for these inputs, implement strategies that minimize the need for PPE, and ensure its proper use.^22^

## OBJECTIVE

The objective of this study was to prove through facts that there were failures in the distribution of PPE to Brazilian medical professionals during the time between February 1 and September 30, 2020.

## METHODS

This research was approved by the Ethics Committee of the Faculty of Medicine of the University of Porto on June 21, 2021, with opinion 04/CEFMP/2021, and its methodology was approved by the coordinator of PhD in bioethics at the same university.

We identified all the institutions representing the class of Brazilian medical professionals.

The first step of the work consisted of prospecting every day, without exception, with respect to the period defined between February 1 and September 30, 2020, of all facts and all news on their official website via the World Wide Web and searching for the complaints of Brazilian doctors regarding the lack of distribution of PPE as part of the fight against COVID-19. These surveys were conducted when these institutions had official websites and/or were available.

The work verified this fact on consecutive days in all 26 states of the federation and in the Federal District, and its sub-regions.

Data were collected from the websites of all official representative entities of physicians, such as CFM and its 27 regional sites, and the AMB and its 27 federated sites. The two union federations, the FENAM, which brings together the 16 State unions—Amazonas (AM), Bahia (BA), Distrito Federal/Capital (DF), Espírito Santo (ES), Goiás (GO), Maranhão (MA), Minas Gerais (MG), Mato Grosso do Sul (MS), Paraná (PR), Piauí (PI), Rio de Janeiro (RJ), Rio Grande do Norte (RN), Rio Grande do Sul (RS), Sergipe (SE), and five other subregional unions (Juiz de Fora and Zona da Mata/MG, Niterói, São Gonçalo and Região/RJ, Norte do Paraná and Santos, São Vicente, Cubatão, Guarujá, and Praia Grande/SP)—the other trade union, the FMB, which brings together eight other remaining States—Acre (AC), Alagoas (AL), Mato Grosso (MT), Pará (PA), Paraíba (PB), Pernambuco (PE), Santa Catarina (SC), and Tocantins (TO), and three subregional States (Anápolis, Campinas and regions, and Southern Region of Santa Catarina)—were all assessed.

This research was complemented by surveying several national and international publications to provide a theoretical basis for this study.

## RESULTS

Table 1 demonstrates the existence of websites of regional representative unions of the Brazilian medical class that have in their own website an announcement about the existence of information on the topic: “Lack of PPE.” They provide a “specific link” for these complaints, which redirects the complaining physician to the AMB form. AMB is the only federal medical institution that provides the sum of these national data on the lack of PPE and identifies the total number of complaints from the states where the doctors’ complaints are coming from. This information was also accessible to the public. In the case of state councils of medicine (CRM), they provide a “specific link” that redirects to the form on the website of the CFM; however, unlike the AMB, they do not have public access, including for doctors. The unions related to FENAM, which do not have their own form, have a link that redirects them to the AMB website. All complaints, including those made directly on the AMB website (https://amb.org.br/epi/), were presented as aggregated data, and there was no individual identification.

**Table 1.** Specific link in website: Lack of personal protective equipment (PPE)

State Unions	DF	BA	MG	PR	PI	RJ	RN	RS
**Specific link**	x	x	x	x	x	x	x	x

DF = Distrito Federal; BA = Bahia; MG = Minas Gerai; PR = Paraná; PI = Piauí; RJ = Rio de Janeiro; RN = Rio Grande do Norte; RS = Rio Grande do Sul.

What we refer to as the “specific link,” is the one that is identified in a specific page in the site of the accessing institution, and that redirects the complaining physician to the site of the AMB, either through its own homepage or even in an internal page with the subtitle of the main menu as the theme “PPE.”

The physicians in states that do not have a specific link for complaints only have the option to do so through the CFM or AMB websites.

The CFM website decided not to present for consultations the totalization of complaints received as complaints of lack of PPE made by physicians.

All state unions that did not have their own website, as well as all those that did, but that did not redirect the physicians’ complaints about the lack of PPE to the AMB or CFM websites, are not part of the data in the tables.

The states of Acre and Alagoas did not have a website available on the World Wide Web at the time.

The states of AM, GO, MG, PR, RS, and MT requested PPE distribution in court. To complain regarding the lack of PPE, it is necessary to be accredited as a doctor at AMB, CFM, and others.

The news published on the official sites of the representative entities of the medical class concerning the PPE theme and the failure in its distribution were all selected one by one, day by day, and identified by accessing their respective sites through menus such as: Home Pages, Publications, News or Dialogues, during the proposed period from January 1 to September 30, 2020 (Table 2). This specific news was encouraged by the involvement of hundreds of complaints on the official websites of their representative institutions whose data were added together and are shown in Figure 2. The data were not included for institutions that did not express specific opinions on the subject, or if the corresponding website was inoperative or non-existent during the period from January 1 to September 30, 2020.

**Table 2.** The news published on the official sites of representative entities

Institutions/ State Unions	CFM	AMB	FMB	DF	BA	AM	ES	GO	MG	PR	PI	RN	RS	SE	SC
**News about the PPE**	**10**	**2**	**6**	**7**	**4**	**8**	**2**	**3**	**19**	**4**	**5**	**3**	**6**	**2**	**1**

PPE = personal protective equipment; CFM = Federal Council of Medicine; AMB = Brazilian Medical Association; FMB = Brazilian Medical Federation; DF = Distrito Federal; BA = Bahia; AM = Amazonas; ES = Espírito Santo; GO = Goiás; MG = Minas Gerais; PR = Paraná; PI = Piauí; RN = Rio Grande do Norte; RS = Rio Grande do Sul; SE = Sergipe; SC = Santa Catarina.

## DISCUSSION

The COVID-19 pandemic has been observed to be a testing time in the area of public health management worldwide, and especially in Brazil. In this context, the numbers of physicians, together with nurses and other health professionals,^23^ are affected along with those of the general public. All these medical professionals are extremely important for the prevention and in the fight against COVID-19. The need to adopt public health policies worldwide has been placed beyond its conventional limits. This fact persisted well beyond the initial three months of this pandemic, which was officially recognized in Brazil by the government and parliament in February 2020 after the publication of a Federal Law.^24^ The frequent lack of PPE numerous times made the medical class struggle to receive them even through lawsuits, such as in the states of AM, GO, MG, PR, RS, and MT. In March 2020, the federal government started releasing extra resources to the states of the federation to combat COVID-19,^25^ and consequently to purchase PPE. Despite the low cost of their acquisition, they were not properly distributed to the Brazilian medical class and other health professionals. The absence of PPE for medical activities can cause increased insecurity and individual stress due to the real increase in the risk of death caused by possible contamination with the SARS-CoV-2 virus, which compromises the second level of Maslow’s scale. Notably, we must never forget the human condition of the physicians.

In this research, specific news about the theme was identified in the official sites of the physicians’ representative institutions, which greatly reinforces the significance of this fact. There were 18 specific news articles from national representative entities, and another 73 news articles, a total of 91 news posts, for their state union representatives only in the research period. During this period, a significant number of physicians made their denouncements, totaling to approximately 4,000 physicians. The denunciations made on the websites of the various state unions and at the federal level were addressed and added to those made directly on the AMB website (Table 2).

## CONCLUSION

The COVID-19 pandemic, which started in 2019 in the People’s Republic of China, spread worldwide and was officially reported in Brazil in February 2020, with its extremely negative aspects becoming more evident in the areas of administrative management involving public health in several countries, including its social and economic consequences. This pandemic also revealed the need for a greater interchange between nations, to stimulate a new proposal to improve the World Health Organization for the practice of data transparency, and for the real need for investments that necessarily transform the management of health services into a higher level and move away from the theme of chronic rhetoric that characterizes some governments. There is an imperative need for more robust national security strategies, as demonstrated by the need to produce our own medical supplies, not depending on other nations for such supplies, efficient logistics to assist in the distribution of equipment and supplies, and so on. Poor management has a negative impact on the QWL of medical professionals. In a global assessment, we cannot forget the human condition of physicians.

Across Brazil, hundreds of complaints about the lack of PPE for the medical class were registered on the websites of representative entities and verified by them. This continued even after the initial phase of the pandemic. Finally, these facts demonstrate the lack of proper management of public services. It was evident that the non-distribution of PPE to health professionals compromised the second level of the Maslow Scale, the level of security, and consequently, the QOL of these professionals. Thus, mysthanasia is a common practice among governments. The states must seek a real reformulation of their posture in relation to bio-laws, which reflect the health of their citizens and health professionals, especially in Brazil, where health is part of the current 1988 Federal Constitution.^26^

## References

[1] Brasil. Ministério da Saúde. Painel Coronavírus (2021). Coronavírus Brasil.

[2] Wikipedia, a enciclopédia livre Pandemia de COVID-19 no Brasil.

[3] Liu YC, Kuo RL, Shih SR (2020). COVID-19: The first documented coronavirus pandemic in
history. Biomed J..

[4] OECD (2020). The impact of the coronavirus (COVID-19) crisis on development
finance. Tackling Coronavirus (COVID-19): Contributing to a global
effort..

[5] United Nations Conference on Trade and Development (UNCTAD) (2020). The Covid-19 shock to developing countries: towards a “whatever
it takes” programme for the two-thirds of the world’s population being left
behind. https://unctad.org/system/files/official-document/gds_tdr2019_covid2_en.pdf.

[6] Ferreira S, Porto D (2019). Mistanásia×Qualidade de vida. Rev Bioetica..

[7] Ferreira S (2019). A mistanásia como prática usual dos governos. J CREMERJ..

[8] Brasil. Ministério da Saúde. (2020). Secretaria de Vigilância em Saúde.

[9] Demografia médica no Brasil (2015). 2015/Coordenação de Mário Scheffer.

[10] Vedovato TG, Andrade CB, Santos DL (2021). Trabalhadores(as) da saúde e a COVID-19: condições de trabalho à
deriva?. Rev Bras Saúde Ocup.

[11] Associação Nacional de Medicina do Trabalho (2020). Brasil ultrapassa a marca de cem médicos mortos por Covid-19,
dois por dia. https://www.anamt.org.br/portal/2020/05/21/brasil-ultrapassa-a-marca-de-cem-medicos-mortos-por-covid-19-dois-por-dia/.

[12] Ferreira A, Demutti CM, Gimenez PEO (2010). A Teoria das Necessidades de Maslow: a influência do nível
educacional sobre a sua percepção no ambiente de trabalho. XIII SEMEAD: Seminários em Administração..

[13] Hesketh JL, Costa MTPM (1980). Construção de um instrumento para medida de satisfação no
trabalho. Rev Adm Empres..

[14] Federação Nacional dos Médicos (2020). Sindicatos..

[15] Associação Médica Brasileira (2020). Faltam EPIs em todo o país.

[16] Federação Nacional dos Médicos (2021). SIMEPAR: Falta condição de trabalho no Paraná..

[17] Conselho Federal de Medicina (2020). Combate à COVID-19: formulário para fiscalização de unidades de
saúde. https://sistemas.cfm.org.br/fiscalizacaocovid/.

[18] Agência Nacional de Vigilância Sanitária (2003). Segurança no Ambiente Hospitalar.

[19] Nguyen LH, Drew DA, Graham MS (2020). Risk of COVID-19 among front-line health-care workers and the
general community: a prospective cohort study. Lancet Public Heal..

[20] Binkley CE, Kemp DS (2020). Ethical Rationing of Personal Protective Equipment to Minimize
Moral Residue During the COVID-19 Pandemic. J Am Coll Surg..

[21] Livingston E, Desai A, Berkwits M (2020). Sourcing Personal Protective Equipment During the COVID-19
Pandemic. JAMA..

[22] Soares SSS, Oliveira Souza NVD, Silva KG (2020). Pandemia de Covid-19 e o uso racional de equipamentos de proteção
individual [Covid-19 pandemic and rational use of personal protective
equipment]. Rev Enferm UERJ..

[23] Ranney ML, Griffeth V, Jha AK (2020). Critical supply shortages: the need for ventilators and personal
protective equipment during the Covid-19 pandemic. N Engl J Med..

[24] BRASIL. Lei N^o^ 13.979, de 6 de fevereiro de 2020 (2020). Dispõe sobre as medidas para enfrentamento da emergência de saúde
pública de importância internacional decorrente do coronavírus responsável
pelo surto de 2019.

[25] Brasil. Controladoria-Geral da União (2020). Portal da Transparência: Execução da Despesa por Programa/Ação
Orçamentária. http://www.portaltransparencia.gov.br/despesas/programa-e-acao?paginacaoSimples=true&tamanhoPagina=&offset=&direcaoOrdenacao=asc&de=01%2F01%2F2020&ate=31%2F07%2F2020&acao=21C0&colunasSelecionadas=linkDetalhamento%2CmesAno%2Cprograma%2Cacao%2CvalorDespesaE.

[26] Gurtler CADS, Corrêa BC, Gurtler MRB, Menezes MSB, Salvetti MCP (2020). Gestão de estoques no enfrentamento à pandemia de
COVID-19. Rev Qual HC..

